# The nutritional status affects the complete blood count of goats experimentally infected with *Haemonchus contortus*

**DOI:** 10.1186/s12917-017-1248-4

**Published:** 2017-11-09

**Authors:** S. Cériac, C. Jayles, R. Arquet, D. Feuillet, Y. Félicité, H. Archimède, J.-C. Bambou

**Affiliations:** 1 URZ, INRA, 97170 Petit-Bourg, Guadeloupe France; 2 PTEA, INRA, 97170 Le Moule, Guadeloupe France

**Keywords:** Blood cells, Gastrointestinal nematode, Goats, Nutrition

## Abstract

**Background:**

Gastrointestinal nematode (GIN) remains the most important pathogenic constraint of small ruminant production worldwide. The improvement of the host immune response against GIN though breeding for improved animal resistance, vaccination and nutritional supplementation appear as very promising methods. The objective of this study was to investigate the effect of four nutritional status differing in protein and energy levels (Hay: 5.1 MJ/Kg of dry matter (DM) and 7.6% of crude protein (CP), Ban: 8.3 MJ/Kg of DM and 7.5% of CP, Soy: 7.6 MJ/Kg of DM and 17.3% of CP, BS: 12.7 MJ/Kg of DM and 7.4% of CP) on the haematological disturbances due to *Haemonchus contortus* infection in Creole kid goats.

**Results:**

No significant effect of the nutritional status was observed for faecal egg count (FEC) but the experimental infection induced haematological disturbances whose intensity and lengthening were dependent on the nutritional status. A transient marked regenerative macrocytic hypochromic anaemia as revealed by a decrease of packed cell volume (PCV), red blood cells (RBC) and hemoglobin and an increase of reticulocytes was observed in all infected groups except Hay. In this latter, the anaemia settled until the end of the experiment. Furthermore, *H. contortus* induced a thrombocytopenia significantly more pronounced in the group under the lowest nutritional status in term of protein (Hay and Ban). A principal component analysis revealed that the variables that discriminated the nutritional status were the average daily gain (ADG) and the PCV, considered as measures of the level of resilience to *H. contortus* infection. Moreover, the variables that discriminated infected and non-infected animals were mostly related to the biology of RBC (i.e. size and hemoglobin content) and they were correlated with FEC.

**Conclusions:**

The severity and the lengthening of the regenerative anaemia and the thrombocytopenia induced by *H. contortus* have been affected by the nutritional status. The protein enriched diets induced resilience to the infection rather than resistance. This suggests that resilience is associated with an improved regenerative capacity of the bone marrow. However, this needs to be further investigated to understand the relationships between resistance, resilience and dietary supplementation.

## Background

Gastrointestinal nematode (GIN) infection remains the most important pathogenic constraint of small ruminant production worldwide. The use of chemical drugs as the unique control method is compromised due to the widespread development of anthelmintic resistant GIN populations throughout the world [1, 2]. In addition, the negative possible environmental impacts of anthelmintic residues and the growing consumer demand for chemical-free animal products increase the need for alternative and/or complementary control strategies [3]. The integrated management of GIN infection aims to better control the GIN populations in order to reach a favourable equilibrium for animal production between host and parasites. Thus, numerous axes of research have been developed to control the parasite population both during the host and the free living stages. The improvement of the host immune response against GIN though breeding for improved animal resistance, vaccination and nutritional supplementation appear as very promising methods [4, 5].

Nutritional management of small ruminants has long been considered as a tool for the control of GIN infections [6, 7]. It has been suggested that the nutritional status and the capacity of the host to mount an efficient immune response against invading pathogens are closely associated [8, 9]. Indeed, mounting an immune response is both expensive, in terms of proteins, and calories, because of the metabolic requirement of immune cells, the synthesis of proteinaceous immune mediators and the repairing of damaged tissue [10]. Minerals, trace elements and vitamins are also required for the development of immunity [11, 12]. However, numerous studies have shown that the level of nutrition of the host improve either the resilience or the resistance to GIN infections [13, 14]. The resistance is considered as the capacity of the host to develop an efficient protective response and limit the level of parasitism and the resilience as the capacity to limit the pathophysiological consequences of the infection and maintain the level of production. The expression of resilience or resistance would depend on the host genotype, the physiological stage and the quality of the diet mainly in term of protein [15, 16].

Studies that investigate the impact of the nutritional status of infected small ruminants on the interaction between the GIN parasitism and the physiological disturbances are lacking. Therefore the objective of this study was to investigate the effect of the nutritional status on the haematological disturbances due to *Haemonchus contortus* infection in Creole kid goats.

## Methods

### Animals, management and experimental design

This experiment was conducted at the Institut National de la Recherche Agronomique Animal Production Unit (INRA-PTEA, Guadeloupe, French West Indies) experimental facilities (16°N16’ latitude, 61°W30’ longitude). The Creole goat kids (*n* = 60, 18.88 ± 3.54 kg body weight (BW); 7 months old) had experienced GIN infection at pasture before been randomly placed indoors in four collective pens (*n* = 15 kids/pen) corresponding to the experimental groups, 1 month before the experimental infection. The animals were drenched with moxidectine (Cydectine®, Fort Dodge Veterinaria S.A., Tours, France, 300 μg/kg BW) and toltrazuril (Baycox Ovis, Bayer HealthCare, Loos, France, 20 mg/kg BW) and housed under worm-free conditions. During this period, nematode faecal egg counts (FEC) remained at zero. Each group was placed under one of 4 distinct dietary status: Hay (Hay ad libitum non supplemented), Ban (Hay ad libitum + 1250 g of Fresh unripe banana/kids), Soy (Hay +250 g Soybean Meal/kids) and BS (Hay ad libitum + 125 g of Soybean Meal +625 g of Fresh unripe banana/kids). The composition and nutritional values of the diets is shown in Table 1. The supplement, fresh banana (cut into thin slices each day) and/or soybean meal were distributed first and individually with the help of yoke traps during the time of diet consumption.. Thereafter, the hay was distributed ad libitum and animals have free-choice access to fresh water. Feeding stalls were long enough to avoid competition for hay between the kids. The offered hay was adjusted to the groups BW (120% of the maximum intake capacity). After a 1 month period of adaptation to the collective pens and the diet conditions, a total of 10 kids/pen in each group were experimentally infected with a single oral dose of 10,000 *H. contortus* third-stage infective larvae (L3) and 5 kids/pen remain non-infected (infected and non-infected groups on the same diet remained in the same collective pen). The L3 were obtained 35 days before challenge from coproculture of monospecifically infected donor Creole goats with isolates previously obtained from Creole goats reared on pasture in different farms in Guadeloupe [17].

**Table 1:** Composition and nutritional values of diets

	Nutritional conditions			
	Hay	Ban	Soy	BS
Ingredients (g/Kg DM)				
Hay	1000	750	780	762.5
Fresh banana	0	250	0	125
Soybean meal	0	0	220	112.5
Chemical composition (%)				
OM^a^	88.2	92.9	94.2	94.7
CP^b^	7.6	8.3	17.3	12.7
NDF^c^	65.9	50.8	46.3	45.0
ADF^d^	32.2	25.8	23.2	21.8
ADL^e^	4.6	3.0	2.2	2.7
ME^f^ (MJ/Kg DM)	5.1	7.5	7.6	7.4

^a^ *OM* Organic Matter, ^b^ *CP* Crude protein, ^c^ *NDF* Neutral Detergent Fiber, ^d^ *ADF* Acid Detergent Fiber, ^e^ *ADL* Acid Detergent Lignin, ^f^ *ME* Metabolizable Energy

### Growth measurements

The animals were weighed weekly from the day of infection until the end of the experiment to adjust the offered quantities at 120% of the maximum intake capacity according to BW changes and to measure the individual growth rates.

### Faecal egg count

Approximately 10 g of faeces were weekly collected during experimental infection directly from the rectum of each animal to determine the FEC. The faeces were kept in plastic tubes to avoid contamination and immediately transported from the experimental facility to the laboratory in refrigerated vials. All samples were individually analyzed using a modified McMaster method for rapid determination and FEC was expressed as the number of eggs/g faeces [18].

### Blood cell counts

During the experimental infection, blood samples were individually collected once a week by jugular venipuncture on each animal by using disposable syringes and 20-Ga needles. A 4 mL portion of each blood sample was placed in commercial anticoagulant tubes (ethylenediamine tetraacetic acid K_3,_ EDTA tubes; Becton Dickinson, Plymouth, UK). Blood samples previously placed in EDTA coated tubes were analyzed for a standard haematological profil using a MS4-e (Melet Schloesing Pharmaceuticals s.a., Rue du Collège 90 CH-2300, La Chaux de Fonds, Suisse). The number of circulating eosinophils was determined according to the method of Dawkins et al. [19] with a Malassez cell counter.

### Calculation and statistical analysis

All the animal variables were analyzed by a linear mixed model using the PROC MIXED of SAS (Version 9, SAS Inst., Inc., Cary, NC, 1999). Because of skewed distributions, FEC and eosinophilia variables were logarithm transformed (ln (FEC + 15), ln (Blood eosinophils + 1) respectively) and the other haematological data were square-root transformed, to normalise residual variances. The model included fixed effects of the days post-infection (T), the dietary status (D), the infection status (I) and the significant interaction between D and I and T as defined below:

$$ {y}_{ijk lm}=\mu +\mathrm{D}i+\mathrm{I}j+{\mathrm{T}}_k+{\left(\mathrm{D}\times \mathrm{I}\times \mathrm{T}\right)}_{ijk}+{a}_{ijl}++{\varepsilon}_{jklm}. $$

where *y* is the observed values; *μ* the overall mean; *D*_*i*_ the fixed effect of the i^th^ dietary condition (*i* = 1 to 4), *I*_*j*_ the fixed effect of the j^th^ infection status (infected vs non-infected), *T*_*k*_ the fixed effect of the k^th^ day post-infection (k = 0 to 49), (D × I × T)_*ijk*_ the interaction of the dietary condition, the infection status and the days post-infection, *a*_*ijl*_ is the random effect associated with the l^th^ animal in dietary condition *i* and infection status *j*; and *Ɛ*_*ijklm*_ the random error. All the interactions were initially tested for all variables and only the (D × I × T)_*ijk*_ was statistically significant (*P* < 0.05) and retained in the model. An unstructured variance-covariance structure was used to model the covariance between two observations on the same animal. The same model was applied for all the animal variables except growth rate (Average Daily Gain, ADG). The ADG of the animals were estimated by adjusting the weight curve with a linear model. Significance was declared at ≤5% of probability. A principal component analysis (PCA) was performed using the FactoMineR package [20] in R version 3.3.2 (R Cor Team, 2016). The PCA allows to explore the relationships between all the variables (zootechnical, parasitological and haematological) and to describe similarities and differences between animals and associations between variables, to better defined the nutritional and the infection status respectively. The PCA was done with the 13 variables (2 parasitological and zootechnical variables and 11 haematological variables) as active variables and the animals as individuals, ignoring the infection status (I) and the dietary conditions (D). The interaction D × I was included in the PCA analysis as illustrative variable to obtain their coordinates on the different principal components. The two first principal components were then used to plot each animal individually from 28 to 49 days post-infection (d.p.i). corresponding to the period of eggs excretion in the faeces.

## Results

### Parasitological and zootechnical parameters

**Fig. 1:**
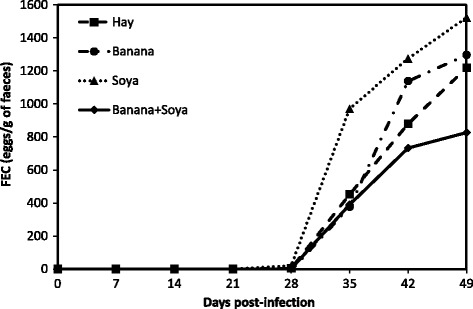
Least square means of Faecal egg counts (FEC) according to the nutritional status: Ban: Banana (•, Infected and gray circle, Non-Infected); Hay (■, Infected and gray square, Non-Infected); Soy: Soybean Meal (▲, Infected and gray triangle, Non-Infected); BS: Soybean Meal + Banana (♦, Infected and gray diamond, Non-Infected)

In the experimentally infected animals, the FEC remained at zero until 28 d.p.i. (Fig. 1). Thereafter FEC increased significantly whatever the dietary status until 49 d.p.i. No significant effect of the dietary status was observed for FEC (*P* > 0.05). No faecal egg excretion was observed in the non-infected animals during the study (data not shown).

**Fig. 2:**
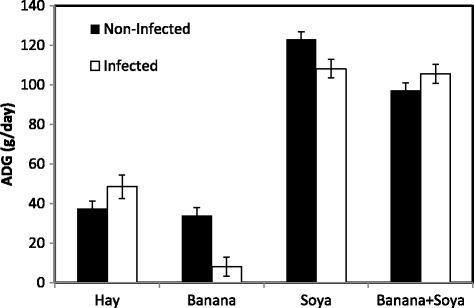
Means and standard errors of Average Daily Gain (ADG) according to the nutritional and the infection status

A significant interaction was observed between the dietary and the infection status for the ADG (*P* < 0.001, Fig. 2). The ADG was significantly higher in the Soy and the BS groups whatever the infection status (*P* < 0.01). No significant difference was observed between the non-supplemented groups (Hay) whatever the infection status and the non-infected kids of the Ban group (*P* > 0.05). A significant negative effect of the infection status was observed only in the Soy and the Ban groups (−12% and −76% between non-infected and infected kids respectively, *P* < 0.05). The ADG of infected kids was significantly higher in the Hay group compared with the Ban group (48.5 vs. 8.05 g/day respectively, *P* < 0.01). No effect of the infection status was observed in the Hay and the BS groups (*P* > 0.05).

### Haematological parameters

**Fig. 3:**
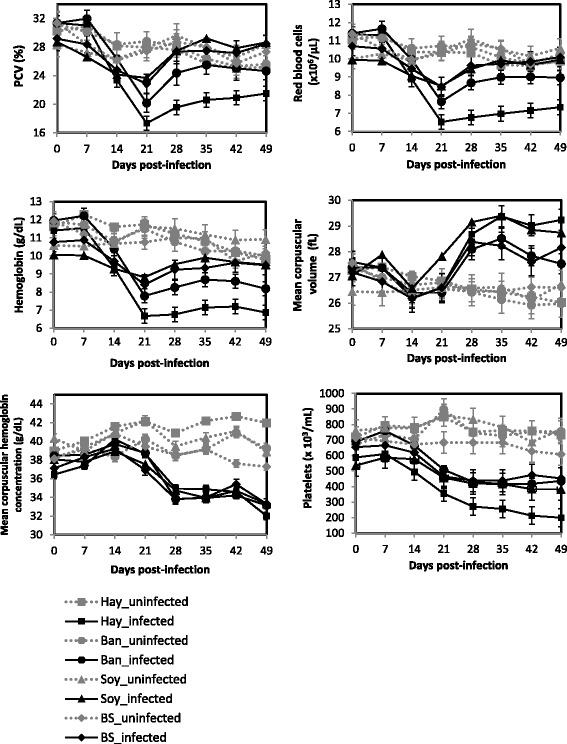
Least square means of haematological parameters according to the nutritional and the infection status: Ban: Banana (•, Infected and gray circle, Non-Infected); Hay (■, Infected and gray square, Non-Infected); Soy: Soybean Meal (▲, Infected and gray triangle, Non-Infected); BS: Soybean Meal + Banana (♦, Infected and gray diamond, Non-Infected)

Significant interactions were observed between the dietary and the infection status and the time (d.p.i.) for all the haematological parameters (*P* < 0.01, Fig. 3). A higher variability of the measured parameters was observed in the infected animals. The packed cell volume (PCV), the red blood cells concentration (RBC), the blood hemoglobin concentration, the mean corpuscular hemoglobin concentration (MCHC) and the platelets concentration significantly decreased in the infected animals but not in the non-infected ones. In the infected animals, at 21 d.p.i. the Hay group reached the lowest values for these haematological parameters except MCHC and it remained significantly lower than the Ban, Soy and BS groups (*P* < 0.01, Fig. 3). The mean corpuscular volume (MCV) increased significantly (*P* < 0.01) in the infected animals whatever the dietary status. No interaction between time and the dietary status was observed for MCV in the non-infected animals (*P* > 0.05).

### Circulating immune cells

**Fig. 4:**
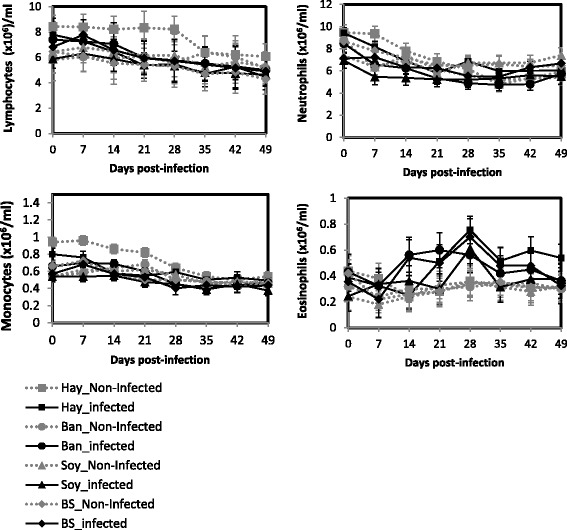
Least square means of blood immune cells according to the nutritional and the infection status: Ban: Banana (•, Infected and gray circle, Non-Infected); Hay (■, Infected and gray square, Non-Infected); Soy: Soybean Meal (▲, Infected and gray triangle, Non-Infected); BS: Soybean Meal + Banana (♦, Infected and gray diamond, Non-Infected)

The results for circulating basophils were negligible whatever the dietary or the infection status (data not shown). A significant interaction between the dietary and the infection status and the time was observed for the circulating immune cells except for circulating monocytes (*P* < 0.01, Fig. 4). Circulating lymphocytes and neutrophils decreased slightly but significantly in infected animals (*P* < 0.05). No variation of the circulating monocytes was observed during the course of the experiment whatever the dietary and the infection status (*P* > 0.05). The circulating eosinophils were significantly higher in the infected animals from 14 to 35 d.p.i. (*P* < 0.001). At 35 d.p.i. the circulating eosinophils decreased to the level of non-infected animals in all dietary status except for Hay.

### Principal component analysis of the haemotological, parasitological and zootechnical parameters

**Fig. 5:**
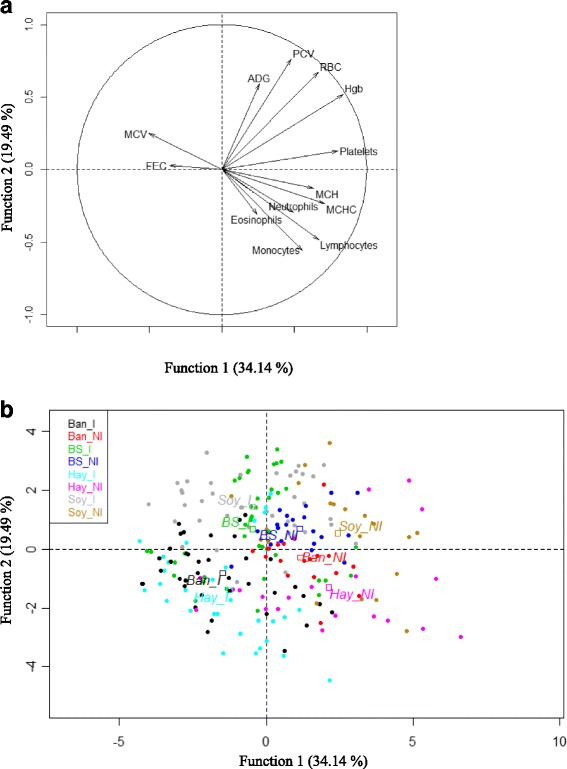
Principal component analysis of the haematological, parasitological and zootechnical parameters. **a** Mapping of the parameters and (**b**) Representation of the animals according to the dietary (Ban: Banana; Hay; Soy: Soybean Meal; BS: Soybean Meal + Banana) and the infection (I: experimentally infected with *Haemonchus contortus*; NI: Non-infected) status from 28 until 49 days post-infection on two-dimensional scatter plot. FEC: Faecal egg counts; MCV: Mean Cell Volume; ADG: Average Daily Gain; PCV: Packed Cell Volume; RBC: Red Blood Cells; Hgb: Hemoglobin; MCH: Mean Corpuscular Hemoglobin; MCHC: Mean Corpuscular Hemoglobin Concentration

The first three principal components of the PCA explained 70.3% of the total variation (34.1%, 19.5% and 16.6% respectively, Table 2). The Fig. 5a shows the contribution of each parameter to the first and the second principal component. The first principal component seemed to contrast FEC, MCV with Platelets, MCH and MCHC. The second principal component seemed to be mainly defined by the contrast between PCV, RBC ADG, and monocytes, lymphocytes and eosinophils. Each animal at each time point, from 28 to 49 d.p.i. was projected on the scatter plot using the two first principal components (Fig. 5b). The infection status (infected and non-infected) was mainly described by the first principal component and the dietary status (Hay, Ban, Soy and BS) by the second principal component.

**Table 2:** Estimate of eigenvalues (λj), percentage of variance (λ%), and cumulative variance and eigenvectors associated to the three first principal components of the animal responses during an experimental *H. contortus* infection (from 28 to 49 days post-infection)

*F* ^a^	λj	λ(%)	Cumulative variance (%)	Associated eigenvectors^b^												
				ADG^b^	FEC^c^	PCV^d^	RBC^e^	Hgb^f^	MCV^g^	MCH^h^	MCHC^i^	Plat^j^	Lym^k^	Mon^l^	Neut^m^	Eos^n^
1	4.78	34.14	34.14	0.26	−0.36	0.48	0.66	0.84	−0.50	0.63	0.71	0.80	0.67	0.55	0.50	0.24
2	2.73	19.49	53.63	0.59	0.03	0.76	0.67	0.52	0.25	−0.13	−0.23	0.13	−0.48	−0.56	−0.29	−0.31
3	2.33	16.63	70.27	0.31	0.31	0.37	0.18	−0.02	0.55	−0.44	−0.61	−0.44	0.54	0.42	0.53	0.16

^a^ *F* Functions (Principal components), ^b^ *ADG* Average Daily Gain, ^c^ *FEC* Feacal Egg counts, ^d^ *PCV* Packed Cell Volume, ^e^ *RBC* Red Blood Cells, ^f^ *Hgb* Hemoglobin, ^g^ *MCV* Mean Corpuscular Volume, ^h^ *MCH* Mean Corpuscular Hemoglobin, ^i^ *MCHC* Mean Corpuscular Hemoglobin Concentration, ^j^ *Plat* Platelets, ^k^ *Lym* Lymphocytes, ^l^ *Mon* Monocytes, ^m^ *Neut* Neutrophiles, ^n^ *Eos* Eosinophils

## Discussion

The nutritional status is closely associated with the intensity of the pathological impact of GIN infection in small ruminants. It has been shown that an improved nutritional status can reduce significantly production losses due to GIN infection by reducing both morbidity and mortality [21, 22]. In *H. contortus* infection, morbidity and mortality are mainly caused by the haematophagous activity of both larval and adult stages of the parasite whose consequence is an irreversible loss of protein. The objective of this study was to investigate the effect of the nutritional status on the haematological disturbances due to *H. contortus* infection in Creole kid goats. No significant effect of the nutritional status was observed for FEC. In accordance with a previous study, a single bolus oral experimental infection of Creole kid goats that previously experienced natural GIN infection at pasture induce a low level of parasitism measured through the FEC and the lengthening of the prepatent period [17]. Nonetheless, we showed a significant interaction between the nutritional and the infection status on the growth rate. The animal placed under the lowest nutritional status in term of protein (i.e. Hay and Ban) showed the lowest growth rate and the experimental infection reduced the growth rate only for animals placed in the Ban and the Soy groups. Furthermore, the experimental infection induced haematological disturbances whose intensity and lengthening were dependent on the nutritional status. A transient marked anaemia as revealed by a decrease of PCV, RBC and hemoglobin concentrations was observed in all infected groups except Hay. In this latter, the anaemia settled until the end of the experiment. Interestingly, the concentration of hemoglobin (MCHC) did not increase proportionally with the increase in RBC size (MCV). These RBC with a larger volume and a lower hemoglobin concentration were probably reticulocytes (i.e. immatures RBC) as previously shown [23]. These results were in accordance with previous studies conducted in sheep, characterizing the type of anaemia induced by *H. contortus* as regenerative macrocytic hypochromic anaemia [24, 25]. Indeed, the pathogenesis of regenerative anaemia includes external hemorrhage which is chronic in the case of *H. contortus* infection. Furthermore, in the present experiment the number of platelets decreased in all infected animals. This thrombocytopenia was significantly more pronounced in the Hay group, as for PCV, RBC and hemoglobin. Similarly, it has been shown in *H. contortus* infected sheep that the number of platelets decreased progressively during the course of *H. contortus* infection [23]. To our knowledge, besides this study in sheep and ours in goats, this result has never been described in the literature. However, a platelet aggregation and adhesion inhibitor from adults *H. contortus* has been identified and characterized in vitro [26]. It must be emphasized that the host blood taken from the abomasal mucosa is the main nutrient source of the parasitic stage of *H. contortus*. Altogether, these results strongly suggested that *H. contortus* has developed a broad-spectrum strategy to manipulate the host’s hemostatic system, which would target especially the blood platelets.

With the exception of eosinophils, the blood leucocytes counts were not affected by the infection and the nutritional status. Indeed, many studies showed significant correlations between resistance/susceptibility to GIN infection and the magnitude of the peripheral blood eosinophil [27]. Even though this relationship has not been observed in all studies, it is largely admitted that peripheral blood eosinophil plays a key role in the protective response to GIN [28–30]. In contrast with a previous study in Creole goat [31], blood eosinophil counts were not affected by the nutritional status. In the present study the nutritional status were not as contrasted as it was previously. On the other hand, it has been shown that peripheral blood lymphocytes are also involved in the protective response to GIN. A significant negative phenotypic correlation has been found between blood lymphocyte counts and *H. contortus* fecundity [32]. In contrast, in another sheep breed, lymphopenia was observed in *H. contortus* infected lambs [33]. However, the close association between the resistance to GIN infection and the host immune response has been demonstrated in a study showing that depletion of CD4+ T lymphocytes significantly increased the parasitic load in a resistant sheep breed [28]. In Creole goats experimentally infected, we have showed that the level of circulating activated CD4+ and CD8+ T lymphocytes were higher in susceptible animals compared with resistant [34]. Altogether these results obtained in different sheep breeds and goats, with different level of parasitism confirmed the effective role of lymphocytes in the host response against GIN, but also emphasize the need to better investigate this relationship.

The comparison between the Hay and the Ban groups showed that infected animals in the Hay groups prioritize growth at the expense of a marked pathological impact while the reverse was observed in the Ban group. These results were partly in keeping with previous studies suggesting that an improved nutritional status (here Soy and BS compared to Hay and Ban) induced resilience rather than resistance to the experimental *H. contortus* infection (i.e. reduced pathological impact for the same parasitic load) [25, 35, 36]. Interestingly, the PCA revealed that in our study the variables that discriminated the nutritional status were ADG and PCV, considered as measures of the level of resilience to *H. contortus* infection, and eosinophils. Moreover, the variables that discriminated infected and non-infected animals at the cellular level were mostly related to the biology of red blood cells (i.e. size and hemoglobin content) and they were correlated with FEC. The association of eosinophils with resilience was not surprising in our animal model since we had previously shown that, in contrast with numerous studies in sheep, blood eosinophil counts were associated with the level of infection rather than the protective response in goats [34].

Otherwise, other studies including one of ours, have also reported resistance to GIN (i.e. reduced pathological impact and parasitic load) induced by an improved nutritional status [31, 37, 38]. Discrepancy between the different studies could be attributed to the animals physiological stage which would influenced the trade-offs between the immune response against invading pathogens and others physiological functions [39]. The quality of the metabolizable protein arising from the rumen (microbial protein vs by-pass protein), could also be an explanation since the amino acid composition of immune proteins is more compatible with that of by-pass proteins [16]. Further research is needed to better understand the nutrition × parasitism interaction in small ruminant for a better fine tuning of the technical recommendations to breeders.

## Conclusions

The results showed that infection with *H. contortus* induced a regenerative anaemia and a thrombocytopenia. The severity and lengthening of these pathological disturbances have been affected by the nutritional status, the protein enriched diets induced resilience to the infection rather than resistance. This suggests that resilience is associated with an improved regenerative capacity of the bone marrow. However, further investigation to understand the relationships between resistance, resilience and dietary supplementation.
